# Analysis of cursive letters, syllables, and words handwriting in a French second-grade child with Developmental Coordination Disorder and comparison with typically developing children

**DOI:** 10.3389/fpsyg.2013.01022

**Published:** 2014-01-20

**Authors:** Caroline Jolly, Edouard Gentaz

**Affiliations:** ^1^Laboratory of Psychology and NeuroCognition - CNRS UMR 5105, University of Grenoble AlpesGrenoble, France; ^2^Psychology of Sensori-Motor, Affective and Social Development Department, Faculté de Psychologie et des Sciences de l'Education, University of GenevaGeneva, Switzerland

**Keywords:** handwriting acquisition, developmental coordination disorder, single case study, comparison, typically developing children

## Abstract

Poor handwriting is a core deficit in Developmental Coordination Disorder (DCD). In a previous study, we compared the evolution of cursive letters handwriting in a girl with DCD throughout her second-grade year with that of typically developing (TD) children. We found that her handwriting evolved much less than that of TD children and remained similar to that of pre-schoolers at all stages, suggesting that her handwriting skills have reached a steady state level. We present here a continuation of this work, in which we focused on the velocity aspects of handwriting in another French child with DCD. Indeed, different velocity patterns have been observed in Chinese and English children with DCD. In the French cursive style of writing, consecutive letters are joined, a major difference with the English script style of writing. We thus analyzed the handwriting of a second-grade French girl with DCD, not only for isolated letters but also for syllables and words, in comparison to that of TD first-graders (6–7 years old; *N* = 85) and second-graders (7–8 years old; *N* = 88). Each written track was digitized, and nine kinematic parameters were measured to evaluate writing fluency. Results showed that the productions of the child with DCD were more similar to those of first-graders than to those of second-graders. In line with our previous study, the most discriminative parameters between the child with DCD and TD children were size and mean speed. Moreover, her handwriting was less fluent than that of TD children. In contrast to previous observations, we observed a higher writing velocity of the child with DCD when compared to TD children, whatever the complexity of the item, and no significant difference with TD children in the pausing time during writing. These differences may reflect linguistic specificities. For syllables and words, each letter was treated separately as a single unit, thus reflecting a problem in anticipation and automation.

## Introduction

Although it seems an easy task for adults, handwriting is in fact a very complex activity. For instance, handwriting requires not only perceptual-motor skills but also cognitive and linguistic skills (Blöte and Hamstra-Bletz, 1991; Viviani, 1994; Chartrel and Vinter, 2004; Vinter and Zesiger, 2007). The letter to be traced and the corresponding movements are intimately related in handwriting activity. Writing a letter requires to retrieve the letter stored in memory, to access the corresponding motor program associated to its tracing, and to execute this program (Ellis and Young, 1988; Van Galen, 1991). Handwriting learning begins in kindergarten, at the age of three, and takes several years before complete acquisition (Zesiger, 1995; Bara and Gentaz, 2007, 2010; Bara et al., 2011). At the beginning, handwriting exercises consist in copying tasks and are very similar to drawing. As learning progresses, writing and drawing activities dissociate, and children learn the visual representations of letters, which are used to guide their production, and the motor programs associated to each one. Handwriting is mastered once it is fully automated. At the cognitive level, the developmental changes in the product and the process of handwriting are associated with a shift from a retroactive control of movement based on feedbacks to a proactive control (Meulenbroek and van Galen, 1988; Zesiger, 1995; Palluel-Germain et al., 2007). Indeed, at the beginning of learning, movements are slow and essentially guided by visual and kinaesthetic feedbacks. With practice, writing becomes automatic and the control of movement is mostly based on an internal representation of motor acts.

Some children never master handwriting despite correct training. Handwriting difficulties in children can be evaluated and diagnosed by using standardized tests such as the BHK (Hamstra-Bletz and Blöte, 1993; Karlsdottir and Stefansson, 2002; Overvelde and Hulstijn, 2011). Handwriting difficulties can be of various origins. Among other possible causes, poor handwriting is a core deficit observed in Developmental Coordination Disorder (DCD) (Miller et al., 2001; Dewey et al., 2002; Smits-Engelsman et al., 2003; Bo et al., 2008; Rosenblum and Livneh-Zirinski, 2008; Cheng et al., 2011). Children with DCD display an atypical motor coordination development (Willoughby and Polatajko, 1995; Dewey and Wilson, 2001; Barnhart et al., 2003; Visser, 2003; Polatajko and Cantin, 2005; Huron, 2011). Five to eight percent of school-age children DCDs are affected by DCDs, with a higher incidence in boys than in girls (2:1) (Mæland, 1992; Wright and Sugden, 1996; Sugden and Chambers, 1998; American Psychiatric Association, 2000; Dewey and Wilson, 2001). The neuroanatomical origins of DCDs are not clear and may be various [for reviews, see Ahonen et al. (2004), Zwicker et al. (2009), Huron (2011)]. Children with DCD are affected in their everyday life and at school, in particular at the level of handwriting, a skill that they barely master (Miller et al., 2001; Geuze, 2005, 2007; Plumb et al., 2008; Chang and Yu, 2010; Huron, 2011). As they might have disorders in automatizing motor movements, each letter is produced by a succession of sequential movements (Mazeau, 1995). Since these movements are under voluntary control, this is extremely costly for the children in terms of attention, and it prevents them from performing higher order academic tasks such as composing or paying attention to the spelling or grammar. In addition, these children have difficulty with online control, i.e., adjusting the motor plan while executing the action, thereby preventing them to shift from a retro-active to a proactive control of handwriting.

Several research studies aiming at describing and understanding handwriting difficulties in children with DCD have been published in the past years. The written productions of children with DCD are of poor quality and usually display erroneous spatial organization (in particular a higher incidence of mirror letters). From a kinematic point of view, these children present a general writing slowness, a slow speed at initiation, an excessive number of unnecessary pen movements, an irregular pressure of the pen on the paper, and a greater variability in time taken and in the form of the letters (Rosenblum et al., 2003; Rosenblum and Livneh-Zirinski, 2008; Chang and Yu, 2010; Jolly et al., 2010; Cheng et al., 2011). It has been recently demonstrated that the general handwriting slowness observed in children with DCD is due to a higher percentage of time spent in pausing, rather than to slow movement execution (Prunty et al., 2013). Moreover, Chang and Yu (2010) showed that children with DCD used a faster stroke velocity than TD children when writing simple characters, but a lower velocity when writing complex characters. It emerges from these studies that children with DCD demonstrate a wide and various spectrum of handwriting difficulties. These inter-individual differences, which reflect the heterogeneity among children with DCD, add another level of complexity to the understanding of the neuro-anatomic bases of the disorder. In this context, an alternative and complementary approach to group studies to provide information relevant to the understanding of cognitive architecture is the single-case analysis (Caramazza, 1986; Caramazza and McCloskey, 1988). We recently provided a longitudinal analysis of the evolution of cursive letter handwriting in a girl with DCD throughout her second-grade year, in comparison with that of pre-school, first-grade and second-grade typically developing (TD) children. We showed that her handwriting only slightly evolved and remained similar to that of pre-schoolers, suggesting that the handwriting skills of this child with DCD have reached a steady state level (Jolly et al., in press). Moreover, we found that the most discriminative kinematic parameters between the child with DCD and TD children were letter size and velocity: She wrote bigger letters, but faster.

In the continuation of this work, we were interested in analyzing the handwriting of a girl with DCD not only of isolated letters, but also of syllables and words in comparison with TD children. More particularly, we were interested in addressing fluency and velocity aspects of her handwriting. Following the observations by Prunty et al. (2013) and Chang and Yu (2010) on velocity features of the handwriting of English and Chinese children with DCD respectively, we thus wondered if these findings could be extended to the Latin based alphabet, and more particularly to the French cursive style of writing in which consecutive letters are joined (Orliaguet et al., 1997; Kandel et al., 2000), a major difference with the English script style of writing. In the Latin alphabetic system, handwriting complexity relates to the length of the item to write rather than to the complexity of letters themselves. To address our question, we thus analyzed the cursive handwriting of a second-grade child with DCD in comparison to those of TD first-graders (6–7 years old; *N* = 85) and second-graders (7–8 years old; *N* = 88), in a task of random dictation of the 26 alphabetic letters, bigrams, trigrams, and small words. Each written track was monitored using a graphic tablet, and nine kinematic parameters were measured to evaluate writing fluency.

## Methods

### Participants

The present study was conducted in accordance with the Declaration of Helsinki. It was approved by the laboratory LPNC ethics committee. It was conducted with the understanding and written consent of each child's parent and in accordance with the ethics convention between the academic organization (LPNC-CNRS) and educational organizations. Concerning the child with DCD, her parents have given written informed consent to publish these case details.

#### Control groups

Eighty-five first-grade children (34 girls) (mean age 6 years and 10 months at the time of the dictations), and 88 second-grade children (43 girls) (mean age 7 years and 11 months at the time of the dictations) participated in the study. None of the children included in the study presented known learning problems or neuromotor disorders. Since the two control groups are the same as those used in our previous study, their characteristics can be found in Jolly et al. (in press).

#### The child with DCD

The child with DCD (L.) is a little girl born in 2002. She was 8 years and 1 month old at the time of the dictations. Early childhood was normal. Graphic and praxic difficulties appeared at the age of four. L. is right-handed and presents a correct tripodic pen holding. She was diagnosed with visuo-spatial dyspraxia (DSM-IV) at the age of six (first-grade) by a neuropsychologist on the basis of a BHK test (Hamstra-Bletz et al., 1987; French version by Charles et al., 2004). Her mean writing speed (number of characters written in 5 min) was 66.6 and was not significantly different from the mean speed of TD first-grade children (48.9 ± 24.4) but L. had a large, irregular, and chaotic handwriting, and problems in spatial organization. Her total score was 30 and differed by 1.5 standard deviations from the mean score of TD first-grade children (13 ± 6.8). No associated disorders have been identified. Before diagnosis, L. has been receiving systematic remediation for graphic activities from the age of 3 to 6 by an occupational therapist (once a week). After diagnosis, she received systematic remediation for graphic activities (three times per week). The remediation that she received used a combination of techniques, including visual-motor training, handwriting practice and also explicit and supplemental handwriting instruction (i.e., a task-oriented approach).

### Task and material for the analysis of written tracks

Children were asked to write, without time limit, the 26 dictated letters, the syllables “be,” “ble,” “bre,” “ch,” “ll,” and “ve,” and the words “cinq,” “dix,” “quinze.” Two dictations for the isolated letters and three dictations for the other items were performed at the end of the school year, in May, within a few days time interval. The items were dictated in four different random orders, and children were asked to write each item once, in cursive. We checked that the dictation order had no effect on children performances (data not shown). Dictations were performed on a sheet of paper placed on a Wacom© Intuos 3 A5 USB graphic tablet (sampling frequency = 5 MHz). All tracks were monitored using specific software (Hennion et al., 2005; Bluteau et al., 2008, 2010; Jolly et al., 2010), which extracts 9 different parameters for each track: (1) “nb strokes” corresponds to the number of pen strokes which constitute the letter; (2) “in-air time” corresponds to the total time (in seconds) during which the pen is not in contact with the tablet; (3) “length” corresponds to the total length of the track in cm; (4) “total time” corresponds to the total writing time in s; (5) “speed” is the mean speed in cm/s (length/time ratio); (6) “nb peaks” corresponds to the number of velocity peaks. The measure of this parameter requires prior filtration of raw data with an order 3 Butterworth filter at a seizure frequency of 8 Hz (Butterworth, 1930); (7) “nb slow mvts” corresponds to the number of slow movements, i.e., group of samples under 150 ms, between which the distance is less than 0.1 cm; (8) “nb pauses” corresponds to the number of pauses, i.e., periods during which the distance is null; (9) “pausing time” corresponds to the total time (in seconds) of pauses.

### Statistical analyses

Mean values and standard deviations were calculated for each letter and each parameter for the two control groups. Comparisons between L. and the normative groups were then performed using an independent samples Student test. The only exception was for the letter “w,” for which only one unique value for each parameter was obtained for L. In this case, the unique value of each parameter was compared to the mean of the different control groups using the *Singlims* software, which was developed by Pr John Crawford's group for the comparison of single case values to a normative group (Crawford and Garthwaite, 2002, 2007; http://www.abdn.ac.uk/~psy086/dept/psychom.htm). In order to counteract the problem of multiple comparisons and to maintain the familywise error rate, a Bonferroni correction was applied for the analysis of isolated letters' tracks. Since 26 comparisons (one per letter) were performed for each parameter, an alpha-correction level of 0.05/26 = 0.0019 was used.

## Results

### Qualitative analysis of the handwritten productions of the child with DCD

**Figure 1:**
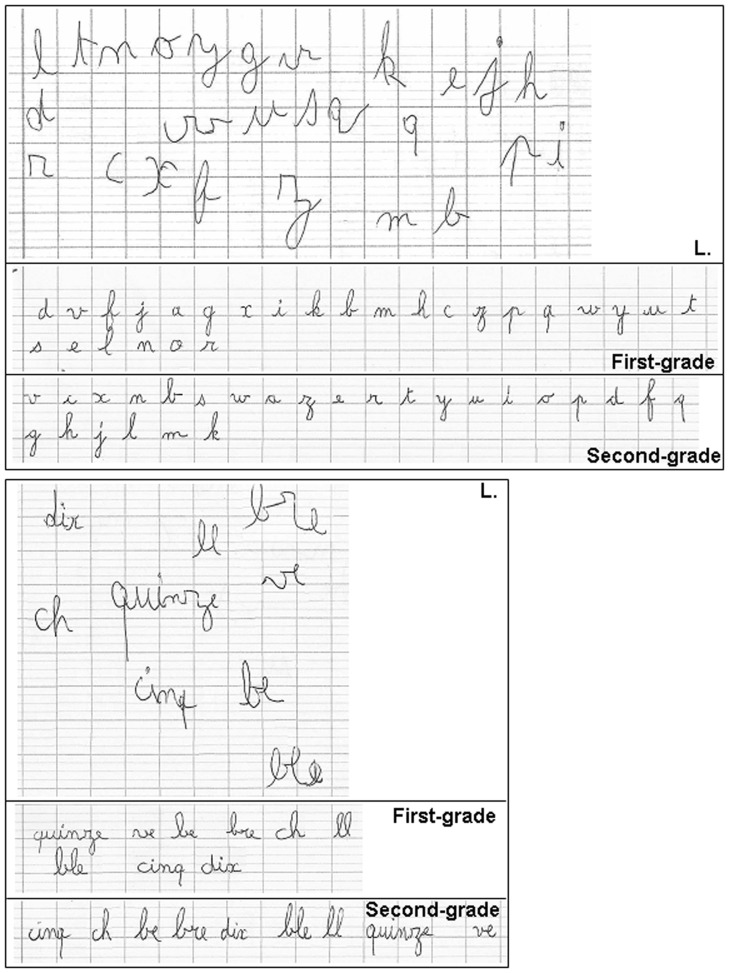
**Samples of cursive handwriting by children.** Examples of dictations performed by the child with DCD, first-graders and second-graders are displayed for isolated letters (**top panel**) and syllables and words (**bottom panel**).

The cursive handwriting of the child with DCD (L.) was analyzed on the basis of a random dictation of the 26 alphabetic letters, 6 bigrams or trigrams, and three words. TD children of first-grade and second-grade performed the same task and served as control groups. Examples of the L's productions and of typical dictations for each control group are presented in Figure 1.

Visually, L's handwriting letters appeared larger than those of both control groups. Moreover, the apparently random position of her productions on the paper sheet and the difficulties she had in following the paper lines suggest problems in spatial organization, a common characteristic of children with DCD (see Introduction).

**Figure 2:**
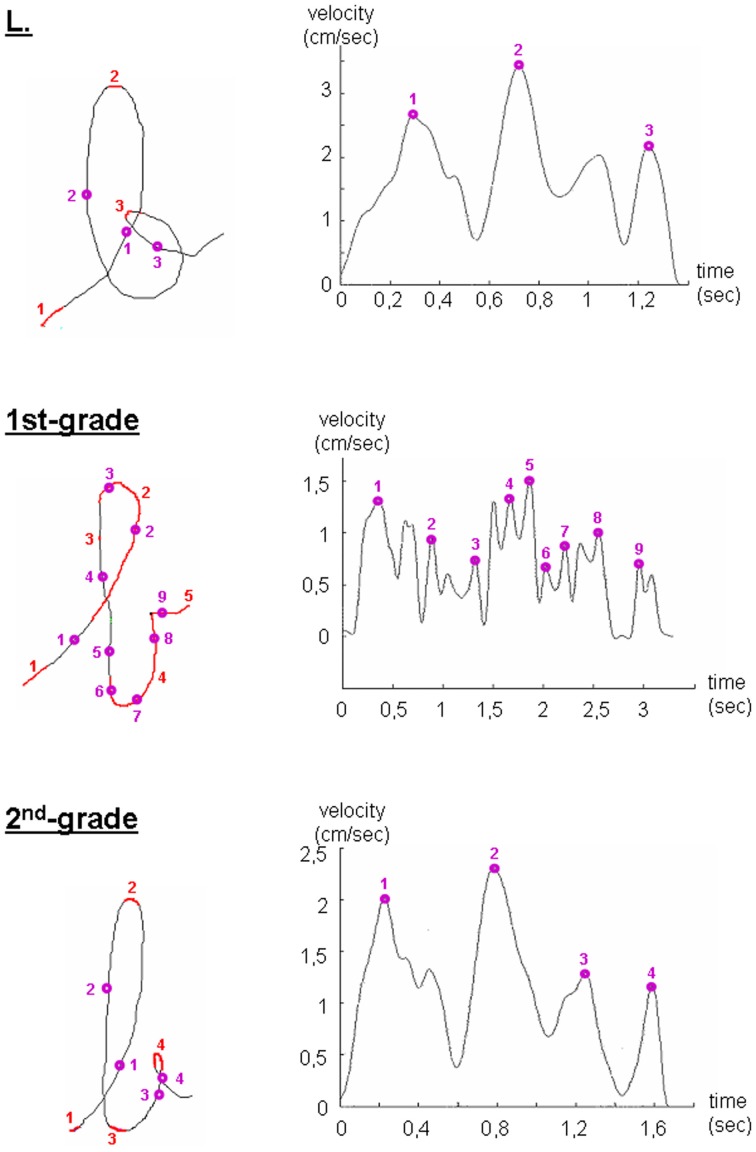
**Examples of the kinematic features of the letter “b” written by the child with DCD (L.) and a random first- and second-grader.** The position of slow moves (red) and velocity peaks (purple circles) are shown on the letter tracks (**right panels**). On the left are shown the corresponding velocity profiles.

**Figure 3:**
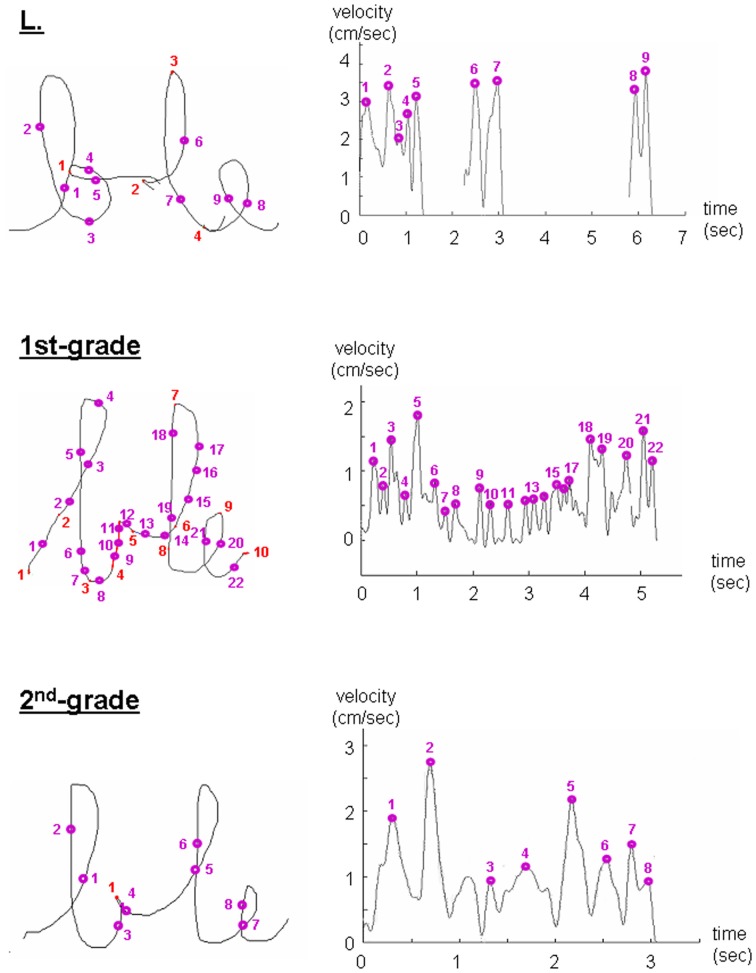
**Examples of the kinematic features of the trigram “ble” written by the child with DCD (L.) and a random first- and second-grader.** The position of slow moves (red) and velocity peaks (purple circles) are shown on the letter tracks (**right panels**). On the left are shown the corresponding velocity profiles.

To further investigate the fluency of L's handwriting from a kinematic point of view, we analyzed the velocity profiles of her written productions as well as those of TD children. Typical examples of a letter and a trigram are presented in Figures 2, 3, respectively. On the left part of the figures are shown the written tracks, on which are indicated the position of slow moves and velocity peaks. On the right are shown the corresponding velocity profiles and velocity peaks. For all items, the velocity profiles of L's productions appeared to be similar to those of second-graders. Likewise, the number and position of velocity peaks and slow moves on the tracks were equivalent to those of second-graders, suggesting a writing fluency similar to her peers. However, the major difference between L. and both first- and graders was the intensity of the velocity peaks on her tracks, which is always higher than that of both first- and second-graders.

### Quantitative analysis of the handwritten productions of the child with DCD

Since we used the same control groups as in our previous study (Jolly et al., in press), we already showed that they all differed from each other.

For each item and each parameter, we compared the results of the child with DCD to those of the two control groups. Tables presenting the values obtained by L. for each item and each parameter, as well as the results of the statistical comparisons between L. and each control group, can be found online in the Supplementary Content. Significant results of these comparisons are presented as follows. Mean values and standard deviations were calculated for each parameter of each item, for the 2 control groups and for the child with DCD. We then compared L's results with those of each control group using a Student test. For isolated letters, due to the huge amount of data generated by our analysis, it was not possible to present a detailed analysis of each parameter, for each item and each group. To facilitate comprehension, we therefore chose to present the results for letters as follows. Firstly, we present a parameter-by-parameter analysis: For each parameter, the number of items for which this parameter was significantly different between L. and the control group (α = 0.0019) is scored. For example, a score of “0” means that no item displayed a different mean for this parameter, i.e., there was no difference between L. and the control group for this parameter. In contrast, a score of “26” for letters for example means that the mean for this parameter was significantly different between L. and the control group for all letters. The higher scores therefore reflect the biggest differences between L. and the group. The scores for the nine parameters are presented altogether in a single graph. Secondly, we performed a letter-by-letter analysis by calculating, for each letter, the number of parameters out of nine which were significantly different between L. and the control group (α = 0.0019). For example, higher scores in the categories “0 or 1 different parameter” mean that there was little to no difference. In contrast, higher scores in the categories “7 to 9 different parameters” reveal strong differences between L. and the group. The overall distribution of these results for the different items is presented in a second graph. To sum up, higher scores reflect a higher number of parameters or items different between L. and the group, and thus a poorer performance of L.

**Figure 4:**
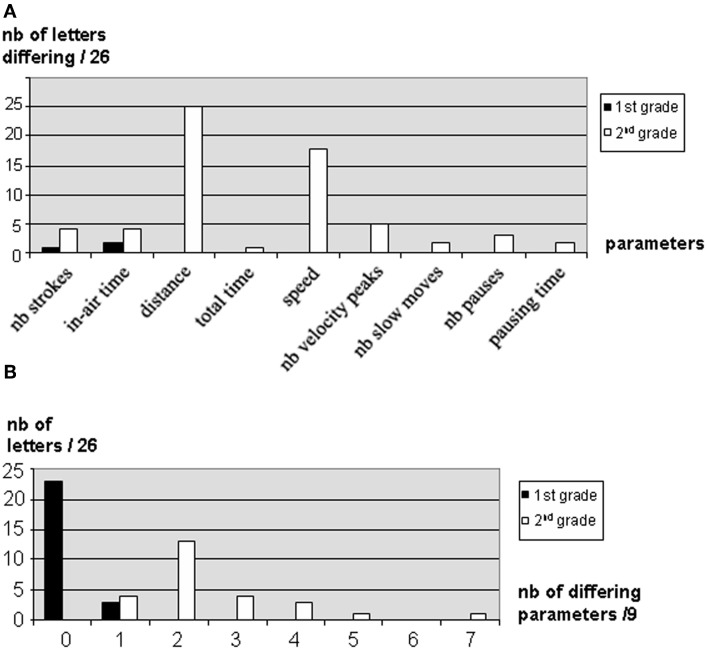
**Comparison between the DCD child's results for isolated letters and those of the 2 control groups.** Results of the DCD child were compared to those of first-graders (black bars) and second-graders (white bars). **(A)** For each parameter, the bars indicate the number of letters for which this parameter is significantly different (α = 0.0019) in the DCD child and the control groups. In **(B)** is presented the histogram of distribution of the differences between the DCD child and the control groups, i.e., the number of letters corresponding to each number of parameters significantly different (α = 0.0019).

In Figure 4 are presented the results of the comparison between the cursive letters produced by L. and those of the control groups. For each parameter, there was a greater difference between L's letters and those of second-graders than those of first-graders (Figure 4A). L's letters displayed very little differences with those of first-graders (mean = 0.11 ± 0.33 parameters different) (Figure 4B). For instance, no difference between L. and first-graders was observed for 23 letters out of 26. In contrast, the overall number of parameters which differed between the child with DCD and the control group was greater for the second-graders' group (Figure 4B). For instance, 22 letters out of 26 displayed at least two different parameters (mean = 2.54 ± 1.36 parameters different). One important observation is that the parameters which were significantly different for the child with DCD always displayed a higher value than the mean of the control group. The most discriminative parameters between the child with DCD and second-graders were track length (25 letters out of 26) and speed (18 letters out of 26): The child with DCD produced larger letters, at a higher speed than TD children of the same age (Figure 4A).

We next compared the results of the child with DCD for bigrams, trigrams and words to those of the two control groups. Tables presenting the values obtained by L. for each item and each parameter, as well as the results of the statistical comparisons between L. and each control group, can be found online in the Supplementary Content.

**Figure 5:**
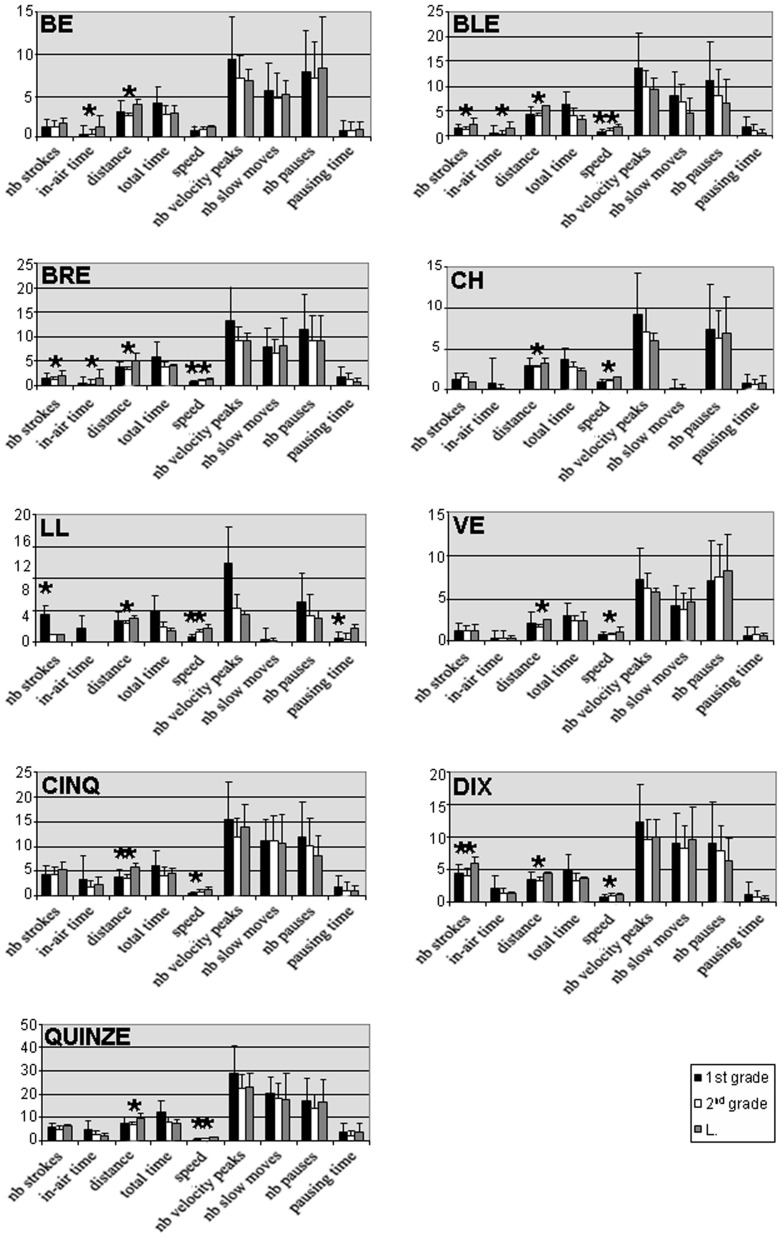
**Comparison between the DCD child's results for syllables and words and those of the 2 control groups.** For each parameter and each item, the mean values and SD were calculated for first-graders (black bars), second-graders (white bars), and for the DCD child (gray bars). Comparisons between L. and each control group were performed by using a Student test. Significant results (*p* = 0.05) are indicated by a star.

For each item and each parameter, we calculated the mean value and the SD for L. and for the two control groups. The means between L. and each control group were compared by using a Student test. Histograms presenting the results are displayed in Figure 5.

As shown in Figure 5, the productions of the child with DCD presented more significant differences with those of TD second-graders than with those of first-graders. For instance, differences with first-graders were only observed in mean speed (7 items), number of strokes (2 items out of 9), distance (1 item), and pausing time (1 item) (mean = 1 ± 0.71). In contrast, differences with second-graders were observed in distance (all items), speed (5 items), number of strokes (3 items out of 9), and in-air time (3 items) (mean = 2.22 ± 1.09). Same as for isolated letters, these results show that syllables and words produced by this second-grade child with DCD are more similar to those of first-graders than to those of second-graders. Again, the child with DCD produces larger items, but at a higher speed. Interestingly, her writing speed is even higher than that of first-graders.

To sum up, our results showed that the handwritten productions of this second-grade child with DCD are more comparable to those of first-graders than those of second-graders, for letters, syllables, and words. Interestingly, the lag between TD children and the child with DCD affected almost all items, even easy or familiar letters such as the “e.”

## Discussion

In the present study, we provide a comparison of the handwritten productions of a second-grade child with DCD with those of TD children of first- and second-grade. This work is a continuation of a recent study presenting a complete 1-year survey of cursive letters produced by a girl with DCD throughout her second-grade year, in comparison to those of TD children of the same age (Jolly et al., in press). In this previous work, we showed that in contrast to TD children, her handwritten productions evolved much less between the end of first-grade and the end of second-grade and remained more similar to those of pre-schoolers, thus showing that the lag between the child with DCD and TD children increased with time, even with remediation, suggesting that she may have reached a steady-state level reflecting his/her maximal writing skills.

In the present work, we were interested in the analysis of fluency and velocity aspects of the handwriting of another second-grade child with DCD. Indeed, Prunty et al. (2013) have showed recently that English children with DCD present a handwriting slowness which is due to an increased time spent in pausing and not to a decreased velocity. In addition, Chang and Yu (2010) reported different velocity profiles in the handwriting of children with DCD depending on the complexity of the Chinese characters to be written. We thus wondered if these findings could be extended to the Latin based alphabet, and more particularly to the French cursive style of writing in which consecutive letters are joined (Orliaguet et al., 1997; Kandel et al., 2000), a major difference with the English script style of writing. Since in the Latin alphabetic system handwriting complexity relates to the length of the item to write rather than to the complexity of letters themselves, we thus analyzed the handwriting of the child with DCD for isolated letters, bigrams, trigrams, and small words, and compared her productions with those of TD first- and second-graders. We found here that all productions of the second-grade child with DCD are more similar to those of first-graders than to those of second-graders. The delay between L. and TD children does not increase importantly with the complexity of the items to be written, suggesting that the treatment of the “letter” unit is the basis of her handwriting difficulties.

From a kinematic point of view, we found that the most discriminative writing parameters between the child with DCD and TD children of the same age were length and velocity: The child with DCD wrote larger but at a higher speed. These results, which are in line with our previous observations on another child with DCD (Jolly et al., 2010), are likely to be due to the principle of isochrony (Binet and Courtier, 1893; Lacquaniti et al., 1983; Wright, 1993). Indeed, it has been shown that there is a proportional and direct relationship between the trajectory length and movement velocity. This invariant feature of handwriting characterizes motor programs in adults. Other handwriting parameters which differentiate the child with DCD from TD children include the number of pen strokes and in-air time, in particular for syllables and words, and the number of velocity peaks for isolated letters. The increased number of pen strokes observed for syllables and words is perfectly illustrated in Figure 3, where it clearly appears that each letter of the trigram is treated separately as a single unit, with a pause in between, thus reflecting a problem in anticipation and automation. Altogether, these observations directly reflect the lesser fluency of the handwriting of the child with DCD. This particular pattern reflects hesitations during handwriting, which may be due to a deficit in procedural memory (Nicolson and Fawcett, 2011). This increased velocity of the child with DCD is likely due to the higher intensity of the velocity peaks during writing, as observed on the velocity profiles.

Our present findings as to the velocity pattern of this DCD child handwriting are distinct from those described by others. In particular, Prunty et al. (2013) observed a slowness in English children with DCD due to increased time spent in pausing, and Chang and Yu (2010) reported various velocity depending on the complexity of the Chinese character to write. In contrast, our results reveal a higher writing velocity of the child with DCD when compared to TD children, whatever the complexity of the item to be written, and no significant difference with TD children in the pausing time during writing. These differences between our results and previous observations may be due to the fact that our analysis is a single-case study while the other studies were group studies. Due to the high heterogeneity among children with DCD, group studies and single-case studies may lead to apparent discrepancies which actually reflect inter-individual differences. These two kinds of approaches are in fact complementary. Group studies reveal general tendencies, while single-case studies allow a detailed analysis of typical or atypical cases (Caramazza, 1986; Caramazza and McCloskey, 1988). Another possible explanation for the apparent discrepancy between our observations and previous ones may relate to the style of writing which was analyzed. Indeed, the French cursive style of writing is quite different from Chinese or Latin script. For instance, Chinese and to a lesser extent script writings require a higher number of strokes than French. Moreover, consecutive letters are joined in French cursive writing, while characters are separated in Chinese or script. Differences between the results of various studies may thus be due to linguistic specificities, as it is the case for example for the learning of reading (Gentaz et al., 2014).

Our present observation that the handwriting of the second-grade child with DCD is similar to that of first-graders is in line with our previous study on another second-grade child with DCD, whose handwriting was closer to that of preschoolers (Jolly et al., in press). Altogether our results support the hypothesis that each child with DCD may reach a steady-state level reflecting his/her own maximal skills on handwriting, and raise again the question of the necessity of handwriting intervention beyond this step.

## Author contributions

Caroline Jolly and Edouard Gentaz designed the project. Caroline Jolly performed the experiments in schools and the statistical analyzes of the results. Caroline Jolly and Edouard Gentaz wrote the paper.

## Conflict of interest statement

The authors declare that the research was conducted in the absence of any commercial or financial relationships that could be construed as a potential conflict of interest.
