# Supplementary material for: Analysis of cursive letters, syllables, and words handwriting in a French second-grade child with Developmental Coordination Disorder and comparison with typically developing children
Source: Front Psychol. 2014 Jan 20;4:1022. doi: 10.3389/fpsyg.2013.01022 (PMC3895814; doi:10.3389/fpsyg.2013.01022)
Supplement: Supplementary file 1 [file DataSheet1.PDF]

Supplementary Figure S1. Comparison of the results of the child with DCD (L.). for cursive letters handwriting with those of first- and second-grade TD children.

For each letter and each parameter, the mean and SD values were calculated for TD children and for the DCD child. The results were compared using an independent sample Student test. A Bonferroni alpha-level correction was adopted (α = .0019). Grey boxes indicate significant differences between L. and the normative group.

		N=		nb strokes	in-air time (sec)	distance (cm)	total time (sec)	speed (cm/sec)	nb velocity peaks	nb slow moves	nb pauses	pausing time
**A**	L.	2	**mean**	2	0.326	1.028	1.025	1.001	2.5	2.5	2	0.147
			**SD**	0	0.122	0.245	0.226	0.018	0.707	3.536	2.828	0.209
	first-grade	85	**mean**	1.381	0.162	1.046	1.311	0.887	3.429	2.583	3.952	0.267
			**SD**	0.579	0.402	0.428	0.555	0.396	1.779	1.499	2.189	0.281
			**p**	0.136	0.566	0.952	0.471	0.688	0.465	0.94	0.218	0.554
	second-grade	88	**mean**	1.376	0.134	0.662	1.133	0.65	2.2	1.447	2.082	0.06
			**SD**	0.556	0.231	0.175	0.472	0.239	0.72	0.716	1.32	0.082
			**p**	0.118	0.246	0.005	0.748	0.043	0.562	0.072	0.932	0.156
**B**	L.	2	**mean**	1	0	2.049	1.365	1.5	3	2.5	3	0.085
			**SD**	0	0	0.449	0.006	0.322	0	0.707	0	0
	first-grade	85	**mean**	1.036	0.043	1.973	2.553	0.849	6.602	3.699	5.747	0.623
			**SD**	0.188	0.342	0.741	0.973	0.38	3.622	1.687	2.942	0.648
			**p**	0.787	0.859	0.179	0.009	0.825	0.051	0.086	0.046	0.212
	second-grade	88	**mean**	1.114	0.17	1.068	1.738	0.663	3.379	2.147	3.256	0.137
			**SD**	0.435	0.649	0.258	0.642	0.239	0.915	1.135	1.763	0.199
			**p**	0.031	0.731	0.382	0.014	0.146	0.001	0.482	0.148	0.522
**C**	L.	2	**mean**	1	0	0.717	0.557	1.332	1	1.5	1.5	0.065
			**SD**	0	0	0.078	0.117	0.421	0	2.121	2.121	0.092
	first-grade	85	**mean**	1.218	0.15	0.717	0.941	0.854	2.355	1.746	3.747	0.23
			**SD**	0.53	0.328	0.442	0.536	0.486	1.537	1.164	2.567	0.256
			**p**	0.448	0.531	0.819	0.302	0.16	0.211	0.787	0.208	0.372
	second-grade	88	**mean**	1.282	0.108	0.384	0.724	0.623	1.282	1.129	1.765	0.055
			**SD**	0.548	0.261	0.113	0.356	0.272	0.548	1.044	1.797	0.114
			**p**	0.471	0.564	0	0.513	0.001	0.471	0.627	0.838	0.904
**D**	L.	2	**mean**	2	0.25	2.233	2.069	1.072	6.5	4.5	5.5	0.32
			**SD**	0	0.029	0.541	0.192	0.162	2.121	3.536	2.121	0.318
	first-grade	85	**mean**	1.562	0.26	1.467	1.806	0.915	4.457	3.266	4.703	0.374
			**SD**	0.848	0.408	0.635	0.905	0.444	2.808	1.881	2.918	0.461
			**p**	0.52	0.949	0.106	0.77	0.614	0.354	0.408	0.772	0.839
	second-grade	88	**mean**	1.494	0.164	0.887	1.258	0.796	2.506	1.541	2.188	0.07
			**SD**	0.59	0.211	0.216	0.523	0.308	0.84	0.933	1.592	0.141
			**p**	0.231	0.565	0	0.032	0.211	0	0	0.005	0.018
**E**	L.	2	**mean**	1	0	1.093	0.973	1.42	2	0.5	2	0.035
			**SD**	0	0	0.11	0.591	0.976	1.414	0.707	1.414	0.042
	first-grade	85	**mean**	1.024	0.01	0.859	1.099	0.922	2.714	1.833	3.75	0.262
			**SD**	0.153	0.068	0.343	0.516	0.521	1.533	1.062	3.143	0.44
			**p**	0.828	0.838	0.34	0.735	0.192	0.516	0.082	0.436	0.47
	second-grade	88	**mean**	1.024	0.02	0.508	0.871	0.702	1.694	1.024	1.576	0.098
			**SD**	0.152	0.169	0.117	0.527	0.301	0.598	0.831	1.614	0.356
			**p**	0.829	0.87	0	0.787	0.002	0.488	0.38	0.714	0.804
**F**	L.	2	**mean**	1	0	2.699	2.022	1.424	6	3.5	5	0.38
			**SD**	0	0	0.53	0.925	0.389	2.828	3.536	5.657	0.474
	first-grade	85	**mean**	1.071	0.088	2.624	2.977	1.04	6.976	4.631	5.857	0.599
			**SD**	0.302	0.666	0.953	1.31	0.543	4.154	2.28	3.09	0.685
			**p**	0.74	0.852	0.912	0.31	0.325	0.743	0.494	0.703	0.655
	second-grade	88	**mean**	1.035	0.009	1.435	2.078	0.8	3.823	2.224	3.306	0.126
			**SD**	0.186	0.05	0.302	0.911	0.32	0.902	1.095	1.718	0.16
			**p**	0.77	0.794	0	0.932	0.008	0.002	0.126	0.196	0.037
**G**	L.	2	**mean**	2	0.391	2.177	1.439	1.517	3.5	2.5	2	0.097
			**SD**	0	0.31	0.151	0.07	0.179	0.707	2.121	1.414	0.117
	first-grade	85	**mean**	1.349	0.119	2.037	2.556	0.925	6.048	3.687	5.554	0.563
			**SD**	0.572	0.207	0.79	1.092	0.484	3.467	1.607	3.109	0.625
			**p**	0.114	0.071	0.804	0.154	0.089	0.305	0.307	0.112	0.298
	second-grade	88	**mean**	1.373	0.102	1.141	1.694	0.756	2.964	1.952	2.855	0.1
			**SD**	0.511	0.158	0.242	0.672	0.274	1.12	1.125	1.958	0.143
			**p**	0.089	0.014	0	0.596	0	0.504	0.504	0.542	0.98
**H**	L.	2	**mean**	1	0	2.248	1.678	1.37	5.5	3.5	5.5	0.292
			**SD**	0	0	0.361	0.513	0.204	2.121	3.536	4.95	0.371
	first-grade	85	**mean**	1.071	0.041	2.082	2.791	0.821	6.69	3.833	6.429	0.719
			**SD**	0.259	0.164	0.83	1.062	0.392	3.267	1.802	3.043	0.741
			**p**	0.699	0.728	0.779	0.145	0.053	0.611	0.8	0.674	0.421
	second-grade	88	**mean**	1.046	0.027	1.164	1.793	0.718	3.514	2.26	3.415	0.192
			**SD**	0.295	0.1	0.32	0.76	0.304	1.169	1.199	2.445	0.353
			**p**	0.789	0.789	0	0.76	0	0.02	0.174	0.274	0.675

**I**	L.	2	**mean**	2	0.256	1.212	1.09	1.121	3	2.5	3	0.18
			**SD**	0	0.04	0.113	0.105	0.212	0	2.121	2.828	0.205
	first-grade	85	**mean**	2.048	0.466	0.945	1.368	0.777	3.964	3.241	5.53	0.39
			**SD**	0.266	0.301	0.45	0.641	0.4	2.233	1.255	2.693	0.432
			**p**	0.8	0.328	0.408	0.544	0.229	0.546	0.417	0.193	0.497
	second-grade	88	**mean**	2.012	0.395	0.478	0.972	0.556	2.341	2.459	3.765	0.113
			**SD**	0.244	0.591	0.155	0.42	0.227	0.733	0.907	1.674	0.084
			**p**	0.946	0.742	0	0.693	0.001	0.21	0.951	0.529	0.284
**J**	L.	2	**mean**	2	0.636	2.558	2.246	1.16	5.5	3.5	3.5	0.212
			**SD**	0	0.533	0.166	0.511	0.19	0.707	4.95	3.536	0.293
	first-grade	85	**mean**	1.916	0.558	1.957	2.668	0.857	6.542	4.928	7.398	0.769
			**SD**	0.356	0.531	0.922	1.33	0.455	4.159	2.383	4.268	0.903
			**p**	0.74	0.837	0.363	0.657	0.352	0.726	0.414	0.205	0.388
	second-grade	88	**mean**	1.943	0.455	1.012	1.615	0.681	3.272	2.827	3.88	0.138
			**SD**	0.437	0.444	0.273	0.625	0.247	0.99	1.131	1.797	0.098
			**p**	0.93	0.596	0	0.179	0.003	0.001	0.477	0.72	0.277
**K**	L.	2	**mean**	1.5	0.158	2.843	2.344	1.218	6	5	5.5	0.297
			**SD**	0.707	0.224	0.551	0.54	0.045	1.414	2.828	2.121	0.343
	first-grade	85	**mean**	1.39	0.417	2.379	4.01	0.665	8.976	4.915	9.232	1.37
			**SD**	0.766	1.289	0.979	1.701	0.343	4.619	2.53	6.481	1.808
			**p**	0.842	0.779	0.508	0.173	0.026	0.368	0.963	0.421	0.407
	second-grade	88	**mean**	1.222	0.155	1.33	2.672	0.566	4.704	3.42	5.148	0.362
			**SD**	0.524	0.468	0.225	1.266	0.195	1.545	1.556	3.218	0.819
			**p**	0.464	0.992	0	0.718	0	0.244	0.166	0.879	0.913
**L**	L.	2	**mean**	1	0	1.744	1.031	1.74	2	1	1.5	0.03
			**SD**	0	0	0.28	0.179	0.574	0	1.414	2.121	0.042
	first-grade	85	**mean**	1.048	0.083	1.832	2.184	1.042	5.357	3.417	5.333	0.528
			**SD**	0.214	0.481	1.924	1.81	1.002	7.335	4.987	3.279	0.775
			**p**	0.755	0.808	0.949	0.373	0.331	0.522	0.498	0.105	0.369
	second-grade	88	**mean**	1.047	0.011	0.896	1.188	0.875	2.141	1.106	1.8	0.056
			**SD**	0.213	0.051	0.197	0.495	0.378	0.538	0.859	1.361	0.12
			**p**	0.757	0.768	0	0.658	0.002	0.713	0.865	0.761	0.766
**M**	L.	2	**mean**	1	0	1.504	1.048	1.453	3.5	2.5	2.5	0.105
			**SD**	0	0	0.285	0.292	0.133	0.707	2.121	2.121	0.134
	first-grade	85	**mean**	1.06	0.098	1.527	2.162	0.76	6.5	3.81	5.774	0.557
			**SD**	0.284	0.509	0.623	0.857	0.324	2.796	2.17	2.69	0.609
			**p**	0.769	0.788	0.96	0.071	0.004	0.135	0.401	0.092	0.3
	second-grade	88	**mean**	1.106	0.035	0.899	1.794	0.543	4.235	2.812	3.518	0.15
			**SD**	0.409	0.147	0.176	0.653	0.16	1.24	0.919	1.637	0.35
			**p**	0.717	0.741	0	0.113	0	0.408	0.645	0.389	0.858
**N**	L.	2	**mean**	1	0	1.241	1.402	1.148	3.5	2	4	0.137
			**SD**	0	0	0.045	0.929	0.793	0.707	0	1.414	0.088
	first-grade	85	**mean**	1.036	0.013	1.091	1.632	0.711	4.452	2.702	5.357	0.443
			**SD**	0.243	0.087	0.444	0.563	0.281	2.02	1.36	2.831	0.481
			**p**	0.837	0.831	0.637	0.574	0.04	0.51	0.47	0.503	0.373
	second-grade	88	**mean**	1.071	0.061	0.645	1.337	0.529	2.953	2.118	3.024	0.114
			**SD**	0.3	0.359	0.166	0.496	0.192	1.133	0.892	1.806	0.21
			**p**	0.742	0.812	0	0.858	0	0.5	0.853	0.451	0.876
**O**	L.	2	**mean**	2	0.916	1.254	1.374	1.029	3	2.5	2.5	0.11
			**SD**	0	0.898	0.102	0.706	0.454	1.414	0.707	0.707	0.049
	first-grade	85	**mean**	1.217	0.1	1.157	1.629	0.816	3.94	2.434	4.614	0.406
			**SD**	0.495	0.263	0.535	0.85	0.435	2.52	1.416	2.603	0.485
			**p**	0.029	0.0001	0.8	0.675	0.495	0.602	0.948	0.257	0.393
	second-grade	88	**mean**	1.176	0.075	0.651	1.172	0.604	2.035	1.235	2.129	0.057
			**SD**	0.413	0.196	0.193	0.39	0.239	0.763	0.984	1.963	0.076
			**p**	0.006	0	0	0.478	0.016	0.085	0.075	0.791	0.332
**P**	L.	2	**mean**	2	0.252	1.68	1.078	1.6	3	4	3	0.09
			**SD**	0	0.046	0.05	0.231	0.388	0	0	1.414	0.021
	first-grade	85	**mean**	1.75	0.356	1.571	2.069	0.88	5.274	4.429	6.631	0.624
			**SD**	0.488	0.334	0.68	0.963	0.458	2.594	1.941	3.966	0.924
			**p**	0.473	0.665	0.821	0.152	0.03	0.221	0.757	0.202	0.419
	second-grade	88	**mean**	1.635	0.213	0.903	1.503	0.701	3.376	3.094	4.082	0.142
			**SD**	0.531	0.238	0.2	0.75	0.268	0.831	1.231	2.083	0.144
			**p**	0.337	0.814	0	0.428	0	0.526	0.304	0.468	0.611
**Q**	L.	2	**mean**	2.5	1.693	1.482	1.086	1.347	3	2.5	3	0.132
			**SD**	0.707	2.118	0.478	0.132	0.276	1.414	2.121	2.828	0.166
	first-grade	85	**mean**	1.524	0.364	1.459	2.071	0.834	4.69	3.321	6.286	0.661
			**SD**	0.649	0.874	0.669	1.168	0.461	3.147	1.629	3.659	0.796
			**p**	0.039	0.042	0.962	0.239	0.122	0.453	0.485	0.212	0.353
	second-grade	88	**mean**	1.524	0.426	0.784	1.513	0.659	2.439	2.488	4.134	0.231
			**SD**	0.593	1.475	0.184	1.01	0.326	1.177	1.091	3.042	0.482
			**p**	0.024	0.236	0	0.555	0.004	0.508	0.988	0.604	0.775
**R**	L.	2	**mean**	1	0	0.869	0.97	0.919	2.5	2.5	4.5	0.225
			**SD**	0	0	0.005	0.212	0.206	2.121	0.707	2.121	0.205

	first-grade	85	**mean**	1.012	0.002	0.988	1.438	0.765	3.929	2.726	5.262	0.372
			**SD**	0.109	0.021	0.413	0.498	0.405	1.861	1.144	2.4	0.328
			**p**	0.878	0.878	0.687	0.191	0.594	0.287	0.782	0.658	0.531
	second-grade	88	**mean**	1.024	0.023	0.552	1.117	0.559	2.729	2.2	3.4	0.128
			**SD**	0.152	0.176	0.133	0.523	0.215	0.822	1.213	2.161	0.221
			**p**	0.829	0.857	0.001	0.694	0.021	0.707	0.729	0.479	0.538
**S**	L.	2	**mean**	1	0	1.134	0.775	1.498	2	1.5	1.5	0.032
			**SD**	0	0	0.122	0.212	0.253	0	2.121	0.707	0.039
	first-grade	85	**mean**	1.06	0.019	0.863	1.245	0.79	3.31	2.202	4.655	0.342
			**SD**	0.238	0.085	0.38	0.604	0.38	2.094	1.19	3.221	0.432
			**p**	0.726	0.749	0.32	0.278	0.011	0.382	0.418	0.172	0.316
	second-grade	88	**mean**	1.059	0.024	0.483	0.967	0.602	2.235	1.776	2.859	0.127
			**SD**	0.237	0.11	0.111	0.571	0.255	0.766	1.073	2.336	0.321
			**p**	0.728	0.759	0	0.638	0	0.667	0.724	0.416	0.681
**T**	L.	2	**mean**	2	0.278	1.576	1.205	1.313	3	3	4	0.18
			**SD**	0	0.012	0.028	0.12	0.107	0	1.414	1.414	0.156
	first-grade	85	**mean**	2.083	0.476	1.433	1.921	0.897	4.774	4.024	6.06	0.513
			**SD**	0.354	0.384	0.523	0.979	0.465	2.49	1.598	3.465	0.746
			**p**	0.742	0.471	0.702	0.306	0.211	0.319	0.372	0.406	0.532
	second-grade	88	**mean**	2.082	0.349	0.815	1.236	0.796	2.929	2.565	3.518	0.166
			**SD**	0.352	0.259	0.185	0.712	0.371	0.997	1.19	2.281	0.386
			**p**	0.743	0.703	0	0.951	0.054	0.921	0.611	0.767	0.959
**U**	L.	2	**mean**	1	0	1.379	1.251	1.115	2.5	3.5	3.5	0.175
			**SD**	0	0	0.114	0.25	0.131	0.707	0.707	0.707	0.099
	first-grade	85	**mean**	1.095	0.065	1.203	1.753	0.773	4.321	3.333	5.81	0.502
			**SD**	0.334	0.266	0.535	0.768	0.411	2.223	1.417	3.114	0.569
			**p**	0.689	0.73	0.645	0.361	0.246	0.253	0.869	0.3	0.422
	second-grade	88	**mean**	1.059	0.036	0.673	1.223	0.611	2.835	2.624	3.471	0.124
			**SD**	0.237	0.17	0.144	0.508	0.225	0.784	0.899	1.9	0.172
			**p**	0.728	0.767	0	0.937	0.002	0.551	0.176	0.983	0.679
**V**	L.	2	**mean**	1.5	0.137	1.312	1.969	0.729	5	4	6.5	0.317
			**SD**	0.707	0.194	0.164	0.713	0.347	0	1.414	0.707	0.18
	first-grade	85	**mean**	1.071	0.241	1.163	1.88	0.697	4.81	2.774	5.69	0.59
			**SD**	0.373	1.969	0.559	0.992	0.37	2.572	1.508	3.383	1.039
			**p**	0.118	0.941	0.708	0.9	0.902	0.917	0.259	0.737	0.713
	second-grade	88	**mean**	1	0	0.603	1.287	0.513	2.776	1.823	2.788	0.097
			**SD**	0	0	0.145	0.517	0.159	0.85	0.953	1.604	0.121
			**p**	0	0	0	0.07	0.067	0	0.002	0.002	0.013
**W**	L.	1		2	1.364	1.95	5.18	0.376	8	6	16	1.865
	first-grade	85	**mean**	1.162	0.086	1.85	3.863	0.524	9.087	4.2	9.8	1.541
			**SD**	0.462	0.297	1.052	1.849	0.272	6.034	2.034	4.56	1.596
			**p**	0.076	0	0.925	0.481	0.591	0.858	0.382	0.18	0.841
	second-grade	88	**MOY**	1.184	0.085	0.93	2.753	0.368	4.579	3.697	6.421	0.445
			**SD**	0.453	0.219	0.208	1.001	0.119	1.602	1.222	2.811	0.475
			**p**	0.074	0	0	0.018	0.941	0.037	0.065	0.001	0.004
**X**	L.	2	**mean**	3.5	0.878	1.012	1.157	0.891	3	3.5	3.5	0.132
			**SD**	0.707	0.086	0.216	0.13	0.287	1.414	3.536	2.121	0.159
	first-grade	85	**mean**	2.072	0.498	1.135	1.591	0.839	3.976	2.988	4.699	0.355
			**SD**	0.342	0.329	0.467	0.718	0.454	2.409	1.444	2.621	0.399
			**p**	0	0.108	0.712	0.398	0.874	0.571	0.632	0.524	0.436
	second-grade	88	**mean**	2.169	0.518	0.696	1.433	0.577	2.542	2.036	3.349	0.191
			**SD**	0.408	0.666	0.185	0.723	0.27	0.954	1.477	2.94	0.395
			**p**	0	0.448	0.019	0.593	0.108	0.507	0.182	0.943	0.835
**Y**	L.	2	**mean**	1	0	2.34	1.861	1.283	4.5	3.5	4.5	0.162
			**SD**	0	0	0.311	0.27	0.353	2.121	0.707	2.121	0.159
	first-grade	85	**mean**	1.072	0.152	2.385	3.298	0.804	8.265	4.554	7.325	0.947
			**SD**	0.304	0.945	1.215	1.749	0.401	6.19	2.416	4.368	1.219
			**p**	0.739	0.822	0.958	0.251	0.098	0.395	0.541	0.366	0.368
	second-grade	88	**mean**	1.077	0.062	1.224	2.1	0.646	3.731	2.949	4.5	0.193
			**SD**	0.313	0.322	0.264	0.729	0.239	1.028	1.183	2.191	0.184
			**p**	0.731	0.787	0	0.647	0	0.309	0.515	1	0.82
**Z**	L.	2	**mean**	1	0	2.728	2.14	1.274	6	4	8	0.375
			**SD**	0	0	0.014	0.035	0.027	0	1.414	2.828	0.247
	first-grade	85	**mean**	1.048	0.042	2.253	3.285	0.75	7.595	4.667	8.095	0.92
			**SD**	0.214	0.222	0.912	1.082	0.361	3.499	1.71	3.269	0.729
			**p**	0.755	0.79	0.467	0.141	0.044	0.523	0.587	0.968	0.299
	second-grade	88	**mean**	1.133	0.078	1.136	2.511	0.52	4.554	3.819	6.072	0.386
			**SD**	0.62	0.362	0.286	1.114	0.226	1.271	1.624	2.686	0.525
			**p**	0,765	0.764	0	0.641	0	0.114	0.877	0.319	0.977

Supplementary Figure S2. Comparison of the DCD child results for bigrams, trigrams and words handwriting with those of first- and second-grade TD children.

For each item and each parameter, the mean and SD values were calculated for TD first-graders, second-graders and for the DCD child (L.). The results were compared using an independent sample Student test. Grey boxes indicate significant differences (p < .05) between L. and each normative group.

		N=		nb strokes	in-air time (sec)	distance (cm)	total time (sec)	speed (cm/sec)	nb velocity peaks	nb slow moves	nb pauses	pausing time
**BE**	L.	3	**mean**	1.667	1.333	3.853	2.952	1.321	6.667	5	8.333	0.923
			**SD**	0.577	1.221	0.742	0.749	0.115	1.528	1.732	6.028	0.914
	first-grade	85	**mean**	1.347	0.396	3.127	4.096	0.856	9.238	5.54	7.833	0.875
			**SD**	0.8	1.094	1.208	1.865	0.412	5.187	3.291	4.85	1.014
			**p**	0.492	0.142	0.3	0.291	0.052	0.392	0.777	0.859	0.935
	second-grade	88	**mean**	1.29	0.288	2.632	2.824	1.028	7.048	4.71	7	0.832
			**SD**	0.611	0.694	0.36	0.959	0.346	2.737	2.95	4.501	0.898
			**p**	0.301	0.016	0	0.821	0.15	0.812	0.867	0.622	0.864
**BLE**	L.	3	**mean**	2.333	1.42	6.026	3.313	1.882	9.333	4.667	6.667	0.59
			**SD**	1.155	1.48	0.072	0.811	0.39	2.517	3.055	4.726	0.81
	first-grade	85	**mean**	1.483	0.6	4.437	6.252	0.796	13.706	8.273	11.223	1.69
			**SD**	0.762	1.458	1.552	2.63	0.369	7.01	4.703	7.686	2.024
			**p**	0.057	0.334	0.078	0.055	0	0.282	0.187	0.307	0.349
	second-grade	88	**mean**	1.262	0.249	4.128	4.199	1.07	9.902	6.918	8.082	1.026
			**SD**	0.545	0.705	0.526	1.3	0.342	3.29	3.607	5.402	1.196
			**p**	0.002	0.01	0	0.249	0	0.77	0.293	0.658	0.536
**BRE**	L.	3	**mean**	2	1.439	5.017	3.868	1.281	9	8	9	0.833
			**SD**	1	1.711	1.439	0.62	0.153	1.732	5.568	5.292	0.686
	first-grade	85	**mean**	1.504	0.575	3.728	5.861	0.715	13.195	7.797	11.327	1.679
			**SD**	0.853	1.239	1.192	2.785	0.298	6.662	3.794	7.233	1.935
			**p**	0.319	0.233	0.064	0.217	0.001	0.278	0.927	0.58	0.451
	second-grade	88	**mean**	1.21	0.305	3.218	3.694	0.942	9.274	6.468	9.113	1.124
			**SD**	0.449	0.824	0.448	1.191	0.289	2.536	2.929	5.109	1.272
			**p**	0.007	0.03	0	0.803	0.049	0.854	0.398	0.97	0.697
**CH**	L.	3	**mean**	1	0	3.337	2.394	1.391	6	0	6.853	0.759
			**SD**	0	0	0.44	0.236	0.067	1	0	4.63	0.929
	first-grade	85	**mean**	1.352	0.763	2.937	3.624	0.916	9.341	0.231	7.396	0.781
			**SD**	0.656	3.068	0.813	1.518	0.4	5.018	0.967	5.556	1.077
			**p**	0.358	0.669	0.4	0.166	0.044	0.255	0.682	0.251	0.331
	second-grade	88	**mean**	1.387	0.179	2.653	2.722	1.041	7.129	0.145	6.371	0.633
			**SD**	0.523	0.454	0.328	0.739	0.304	2.883	0.438	3.345	0.584
			**p**	0.208	0.5	0.001	0.449	0.052	0.504	0.571	0.173	0.284
**CINQ**	L.	3	**mean**	5.333	2.353	5.694	4.568	1.318	14	10.667	8	1.103
			**SD**	1.528	1.643	0.98	1.07	0.463	4.583	5.859	4.359	0.929
	first-grade	85	**mean**	4.512	3.445	3.915	6.273	0.707	15.476	11.127	11.711	1.885
			**SD**	1.564	4.726	1.487	2.923	0.324	7.397	4.453	7.268	2.341
			**p**	0.368	0.691	0.041	0.316	0.002	0.731	0.86	0.38	0.565
	second-grade	88	**mean**	4.406	1.875	3.757	4.273	0.969	11.922	11.031	10.125	1.251
			**SD**	1.388	1.242	0.619	1.569	0.333	3.819	5.077	5.596	1.426
			**p**	0.264	0.522	0	0.749	0.086	0.364	0.904	0.52	0.86
**DIX**	L.	3	**mean**	6	1.382	4.381	3.577	1.23	10	9.667	6.333	0.497
			**SD**	1	0.177	0.201	0.302	0.113	2.646	5.033	3.512	0.45
	first-grade	85	**mean**	4.353	2.196	3.407	4.844	0.801	12.377	9.066	8.982	1.122
			**SD**	1.358	1.906	1.286	2.437	0.356	5.739	4.527	6.409	1.875
			**p**	0.038	0.462	0.193	0.37	0.039	0.476	0.82	0.477	0.565
	second-grade	88	**mean**	4.048	1.259	3.264	3.358	1.051	9.661	8.242	7.919	0.805
			**SD**	1.179	0.892	0.509	1.047	0.324	2.958	3.574	3.725	0.831
			**p**	0.007	0.813	0	0.721	0.348	0.847	0.509	0.473	0.528
**LL**	L.	3	**mean**	1	0	3.633	1.722	2.18	4.333	0	3.667	2.18
			**SD**	0	0	0.355	0.377	0.528	0.577	0	1.155	0.528
	first-grade	85	**mean**	4.353	2.196	3.407	4.844	0.801	12.377	0.359	6.207	0.678
			**SD**	1.358	1.906	1.286	2.437	0.356	5.739	1.743	4.749	0.836
			**p**	0.038	0.462	0.193	0.37	0.039	0.476	0.722	0.362	0.003
	second-grade	88	**mean**	1	0	2.92	2.291	1.425	5.413	0.143	3.984	0.424
			**SD**	0	0	0.523	0.888	0.508	2.099	0.592	3.457	0.762
			**p**	1	1	0.023	0.276	0.014	0.381	0.68	0.875	0.493
**QUINZE**	L.	3	**mean**	6.333	2.226	9.504	7.355	1.315	23.333	17.667	16.463	3.721
			**SD**	0.577	1.116	2.127	1.782	0.246	5.508	11.015	9.973	3.785
	first-grade	85	**mean**	5.629	4.937	7.64	12.12	0.696	28.901	20.056	16.914	3.797
			**SD**	2.088	3.765	2.729	4.731	0.3	11.67	7.311	10.157	3.809
			**p**	0.562	0.216	0.242	0.084	0.001	0.412	0.579	0.266	0.297
	second-grade	88	**mean**	4.742	2.595	6.994	8.001	0.92	22.5	18.452	14.113	2.386
			**SD**	1.609	1.815	0.923	2.035	0.23	5.933	5.944	5.851	1.975
			**p**	0.095	0.73	0	0.592	0.005	0.813	0.83	0.28	0.442

**VE**	L.	3	**mean**	1.333	0.251	2.496	2.419	1.128	5.667	4.667	8.333	0.565
			**SD**	0.577	0.435	0.097	0.908	0.384	0.577	1.528	4.041	0.403
	first-grade	85	**mean**	1.325	0.302	2.12	3.083	0.75	7.187	4.092	6.95	0.702
			**SD**	0.711	0.894	1.263	1.422	0.364	3.62	2.51	4.871	0.938
			**p**	0.984	0.922	0.608	0.421	0.075	0.469	0.693	0.625	0.801
	second-grade	88	**mean**	1.258	0.333	1.662	2.348	0.742	6.129	3.694	7.403	0.823
			**SD**	0.477	0.931	0.267	0.601	0.182	1.806	1.887	3.907	0.718
			**p**	0.792	0.88	0	0.845	0.001	0.662	0.384	0.689	0.54
